# Pharmacokinetics and pharmacodynamics of drug‒drug interactions in hospitalized older adults treated with direct oral anticoagulants

**DOI:** 10.1007/s40520-024-02768-w

**Published:** 2024-05-22

**Authors:** Théodore Decaix, Kenza Kemache, Pierre Gay, Flora Ketz, Olivier Laprévote, Éric Pautas

**Affiliations:** 1 Geriatrics department, APHP Paris Cité University, Lariboisière-Fernand Widal Hospital, Paris, France; 2 Paris-Cité University, CNRS, Paris, F-75006 CitCoM France; 3 Acute Geriatrics Unit, Charles Foix Hospital, APHP Sorbonne University, Ivry-sur-Seine, France; 4 Department of biology, National Hospital Center Of ophthalmology, 15-20, F-75012 Paris, France; 5 Therapeutic innovations in hemostasis, Paris-Cité University, UMR-S 1140, Inserm, Paris, France; 6 Medical school, Sorbonne University, Paris, France; 7 Faculty of Pharmacy, Paris-Cité University, 4 avenue de l’Observatoire, Paris, 75006 France

**Keywords:** Older adults, Drug-drug interaction, Direct oral anticoagulant, CYP3A4, P-glycoprotein

## Abstract

**Purpose:**

Polypharmacy is a frequent situation in older adults that increases the risk of drug-drug interactions (DDIs), both pharmacokinetic (PK) and pharmacodynamic (PD). Direct oral anticoagulants (DOACs) are frequently prescribed in older adults, mainly because of the high prevalence of atrial fibrillation (AF). DOACs are subject to cytochrome P450 3A4 (CYP3A4)- and/or P-glycoprotein (P-gp)-mediated PK DDIs and PD DDIs when co-administered with drugs that interfere with platelet function. The aim of our study was to assess the prevalence of DDIs involving DOACs in older adults and the associated risk factors at admission and discharge.

**Methods:**

This was a cross-sectional study conducted in an acute geriatric unit between January 1, 2018 and December 31, 2022, including patients over 75 years of age treated with DOACs at admission and/or discharge, for whom a comprehensive collection of co-medications was performed.

**Results:**

From 909 hospitalizations collected, the prevalence of PK DDIs involving DOACs was 16.9% at admission and 20.7% at discharge, and the prevalence of PD DDIs was 20.7% at admission and 20.2% at discharge. Factors associated with DDIs were bleeding history [adjusted odds ratio (ORa) 1.74, 95% confidence interval (CI) 1.13–2.68], number of drugs > 6 (ORa 2.54, 95% CI 1.88–3.46) and reduced dose of DOACs (ORa 0.39, 95% CI 0.28–0.54) at admission and age > 87 years (ORa 0.74, 95% CI 0.55–0.99), number of drugs > 6 (ORa 2.01, 95% CI 1.48–2.72) and reduced dose of DOACs (ORa 0.41, 95% CI 0.30–0.57) at discharge.

**Conclusion:**

This study provides an indication of the prevalence of DDIs as well as the profile of DDIs and patients treated with DOACs.

## Introduction

Polypharmacy is a prevalent medical condition among the elderly worldwide [1]. Although a threshold of 5 or 10 concurrently prescribed medications is often used to define polypharmacy, there is no consensus on its definition [2]. A simple definition that is commonly used is “the administration of more medicines than is clinically indicated, indicating unnecessary drug use” [3]. However, polypharmacy increases the risk of drug–drug interactions (DDIs), regardless of PK or PD [1]. These DDIs can lead to interindividual variability and result in severe adverse drug effects, highlighting the importance of detection and monitoring [1].

Cytochrome P450 3A4 (CYP3A4) and P-glycoprotein (P-gp) are phase I enzymes and phase III efflux transporters, respectively, that are particularly involved in DDIs because of the number of medications in the market on which they have an impact [4, 5]. The prevalence of DDIs involving these two proteins is significant among older adults and is correlated with the number of concurrently prescribed medications [6]. Certain cardiovascular drugs, central nervous system agents, and chemotherapy regimens can cause adverse effects with clinical implications [6].

Direct oral anticoagulants (DOACs) are commonly prescribed medications in older adults because of the high prevalence of atrial fibrillation (AF) in this population [7]. It is prescribed for nonvalvular AF to prevent thromboembolic risk. In addition to age, weight, and renal function, DDIs can contribute to interindividual variability in DOAC treatment response [8–11]. These interactions may involve PK through CYP3A4 and P-gp and PD via co-administration of drugs that interfere with platelet function [12]. Serious adverse effects, such as bleeding or therapeutic failure, such as thrombosis, can result from these interactions [13]. Therefore, anticoagulants are associated with a significant iatrogenic risk, which highlights the importance of close monitoring of their prescription.

In 2019, Gallo et al. measured the prevalence of DDIs involving CYP3A4 and P-gp in a cohort of 3,803 patients aged > 65 years hospitalized for acute events [6]. The prevalence of CYP3A4 and P-gp was found to be approximately 8–11% and 2–4%, respectively, predominantly involving cardiovascular medications and those targeting the central nervous system [6]. However, DOACs were underrepresented in this study, preventing a specific analysis of DDI. Given the clinical impact of potential DDIs involving DOACs, our study was designed to specifically analyze the prevalence and factors associated with PK DDIs targeting CYP3A4, P-gp, and PD DDIs within a population of older adults treated with DOACs.

## Materials and methods

We conducted a cross-sectional study based on retrospective prescreening of patients admitted to the acute geriatric unit at Charles Foix Hospital (AP-HP) between January 1, 2018, and December 31, 2022. All patients hospitalized during this period were eligible. The screening process was performed using the hospital’s information system, which is routinely managed by the health care staff for the financing of hospital activities (Programme de Médicalisation des Systèmes d’Information - PMSI). Among these patients, those who were prescribed DOACs upon admission or at the time of hospital discharge could be included. All separate hospitalizations at least 1 year apart for the same patient were included in the study. Patients who did not meet this criterion were excluded from the study. Patients < 75 years of age, in-hospital deaths, and transfers to another care unit were also excluded from the study.

The endpoint corresponded to the prevalence of co-administration of medications with PK DDIs involving CYP3A4 and/or P-gp or PD DDIs. The drugs were determined from the references listed below [14–17]. The most frequently interacting drug combinations were extracted as those with a relative frequency > 5%. Some drugs were grouped by class when considered relevant, including azole antifungal agents (ketoconazole, itraconazole, posaconazole and fluconazole), calcium channel blockers (verapamil and diltiazem), macrolides (erythromycin and clarithromycin), SRIs (fluoxetine, paroxetine, citalopram, escitalopram, sertraline, venlafaxine, duloxetine) and antiplatelet agents (acetyl salicylic acid, clopidogrel and ticagrelor). General characteristics such as age, sex, weight, Charlson Comorbidity Index (CCI), and Activity of Daily Living (ADL) corresponded to patient data at the time of admission to the unit. Cognitive impairments were defined on the basis of their description in the patient’s medical records. Serum creatinine level was the nadir during hospitalization. Creatinine clearance (ClCr) was calculated using the Cockcroft formula, which is important for drug dosage adjustment, and glomerular filtration rate (GFR) was calculated using the CKD-EPI formula, allowing for a more accurate estimation of renal function. All these clinical and biological data are routinely collected in the unit. The type of DOAC used and the prescribed dosage (full or reduced) were reported. DOAC on the patient’s usual prescription defined DOAC at admission, and DOAC on the discharge prescription issued by the clinician in charge of the patient during hospitalization defined DOAC at discharge. The prevalence of DDIs was measured at admission for patients with a DOAC at admission and at discharge for patients with a DOAC at discharge whether or not they had a DOAC at admission. A history of bleeding events under DOAC treatment was also specified. Bleeding history was classified according to the International Society on Thrombosis and Hemostasis (ISTH) criteria, including major, clinically relevant nonmajor, and minor bleeding [18, 19].

The number of medications administered at admission corresponded to the patient’s usual prescription by their treating physician. The number of medications at discharge was the prescription provided by the geriatrician in charge of the patient upon hospital discharge. This number of medications excluded vitamins, laxatives, and conditionally prescribed medications. When the term polypharmacy was used, this corresponded to a higher than mean number of drugs prescribed in the study population.

Data are presented as the mean standard deviation (SD), median with interquartile range (IQR), or number with percentage as appropriate. The chi-square test or Fisher’s exact test was performed for the comparison of categorical variables. Univariate logistic regression analysis was conducted for all variables. Only variables with *p* < 0.1 were included in the multivariate logistic regression model to avoid the risk of overestimation [20, 21]. All statistical analyses were performed using R software (package questionr). Figure 2 was generated using GraphPad Prism 9.0.0 software (GraphPad, GraphPad Software, La Jolla, CA).

## Results

### Description of the study population

**Fig. 1:**
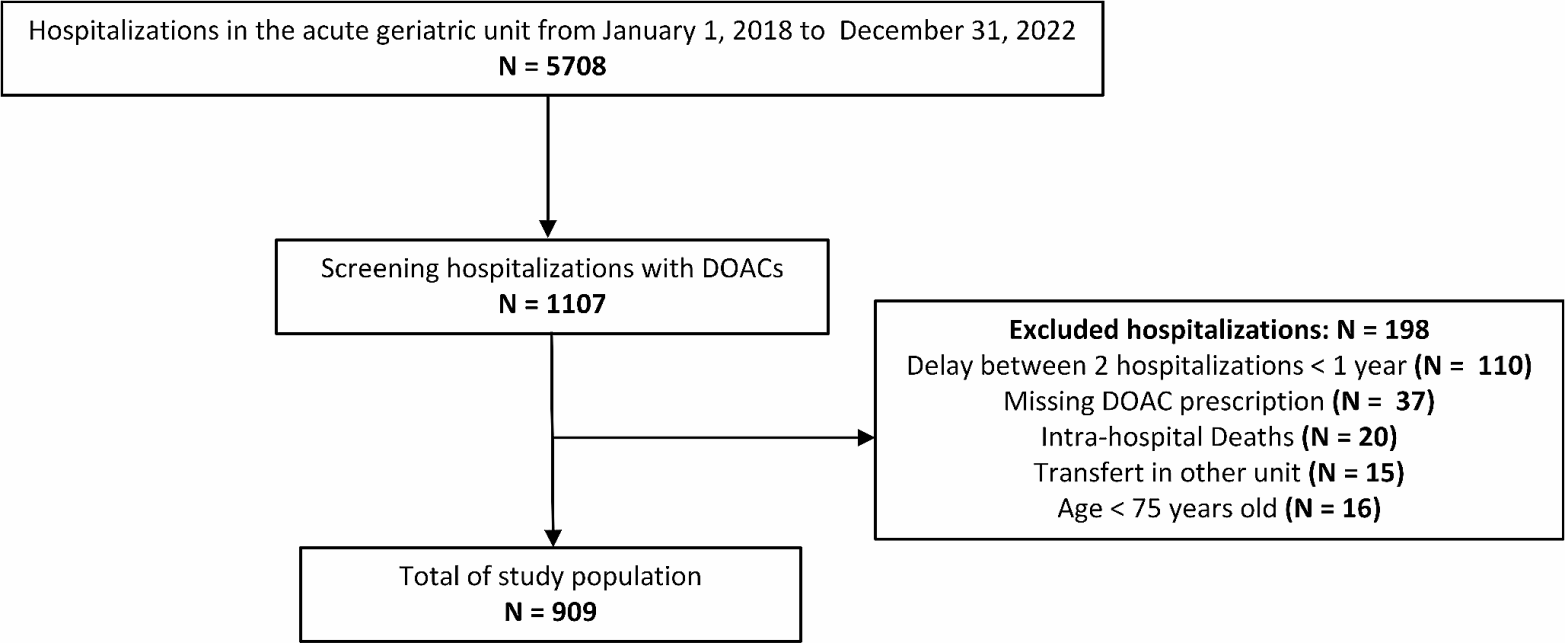
Flow chart. Abbreviation: DOAC: Direct Oral Anticoagulant

From a screening of hospitalizations between January 1, 2018 and December 31, 2022 (*N* = 5708), 1107 hospitalization records mentioned the use of DOACs, from which 198 hospitalizations were excluded, resulting in 909 hospitalizations retained for the final analysis (Fig. 1). Forty-six patients were included multiple times in the analysis when separate hospitalizations occurred more than one year apart for the same patient.

The baseline characteristics of the study population described in this section are shown in Table 1.

**Table 1:** Baseline characteristics

	*N* = 909	NA (%)
Sex (male)	297 (32.7)	
Age (years)	87.2 ± 5.7	
ADL	5 (3.5–6)	
Charlson comorbidity index	7 (6–8)	
Length of stay (days)	14.8 ± 8.0	
Cognitive impairment	526 (57.9)	
Weight (kg)	64.3 ± 15.3	
Serum albumine level (g/L)	33.4 ± 5.4	7 (0.8)
Serum creatinine level (µM)	87.0 ± 34.3	3 (0.3)
ClCr (mL/min)	47.6 ± 19.5	3 (0.3)
GFR (mL/min/1.73m^2^)	61.4 ± 17.8	3 (0.3)
Bleeding history		
*Total*	109 (12.0)	
*Gastrointestinal*	41 (4.5)	
*Intracranial*	16 (1.8)	
*Hematuria*	14 (1.5)	
*Hemoptysis*	2 (0.2)	
*Deep hematoma*	25 (2.7)	
*Epistaxis*	4 (0.4)	
*Deglobulization*	7 (0.8)	
Indication of DOAC		
*AF*	768 (84.5)	
*VTE*	123 (13.5)	
*AF + VTE*	18 (2.0)	
Number of drugs at admission	6 (4–7)	
Number of drugs at discharge	5 (4–7)	

The data presented as mean ± standard deviation, median (interquartile range), and number (percentage)

NA represented missing data

Abbreviations: ADL: Activities of Daily Living. AF: Atrial fibrillation. ClCr: creatinine clearance. DOAC: Direct oral anticoagulant. GFR: Glomerular Filtration Rate. VTE: Venous thromboembolism

The mean age was 87.2 ± 5.7 years, with 32.7% males. The median ADL and CCI were 5 (IQR 3–6) and 7 (6–8), respectively. The mean length of stay was 14.8 ± 8.0 days, and 57.9% of patients were cognitively impaired. The mean weight and serum albumin were 64.3 ± 15.3 kg and 33.4 ± 5.4 g/L, respectively. The means for serum creatinine, ClCr with the Cockcroft formula, and GFR with the CKD-EPI formula were 87.0 ± 34.3 µM, 47.6 ± 19.5 mL/min and 61.4 ± 17.8 mL/min/1.^73 m2,^ respectively. 12% of patients had a history of bleeding. The indications for prescribing DOACs were 84.5% AF, 13.5% VTE, and 2.0% double indication (AF + VTE). The median number of drugs was 6 (IQR 4–7) and 5 (IQR 4–7) at admission and discharge, respectively. The medication data of the study population are presented in Table 2.

**Table 2:** Medications datas

	At admission	At discharge
DOAC	682 (75.0)	798 (87.8)
Type of DOAC		
*Apixaban*	444 (48.8)	593 (65.2)
*Rivaroxaban*	213 (23.4)	205 (22.5)
*Dabigatran*	25 (2.7)	8 (0.9)
DOAC dosage		
*Full dose*	251 (27.6)	305 (33.5)
*Reduced dose*	658 (72.4)	604 (66.5)
PK DDIs	154 (16.9)	188 (20.7)
PD DDIs	190 (20.9)	184 (20.2)

The data presented as number (percentage)

Abbreviations: DDIs: drug-drug interactions. DOAC: Direct oral anticoagulant. PK: pharmacokinetic. PD: pharmacodynamic

DOACs were prescribed for 682 hospitalizations at admission versus 798 at discharge. Apixaban was prescribed by 48.8% of patients, compared with 23.4% for rivaroxaban and 2.7% for dabigatran at admission versus 65.2%, 22.5% and 0.9% at discharge. Reduced dose prescriptions were present in 72.4% of patients at admission compared with 66.5% at discharge. There were 16.9% PK DDIs at admission compared with 20.7% at discharge and 20.9% PD DDIs at admission compared with 20.2% at discharge.

### Distribution of drugs involved in DDIs

**Fig. 2:**
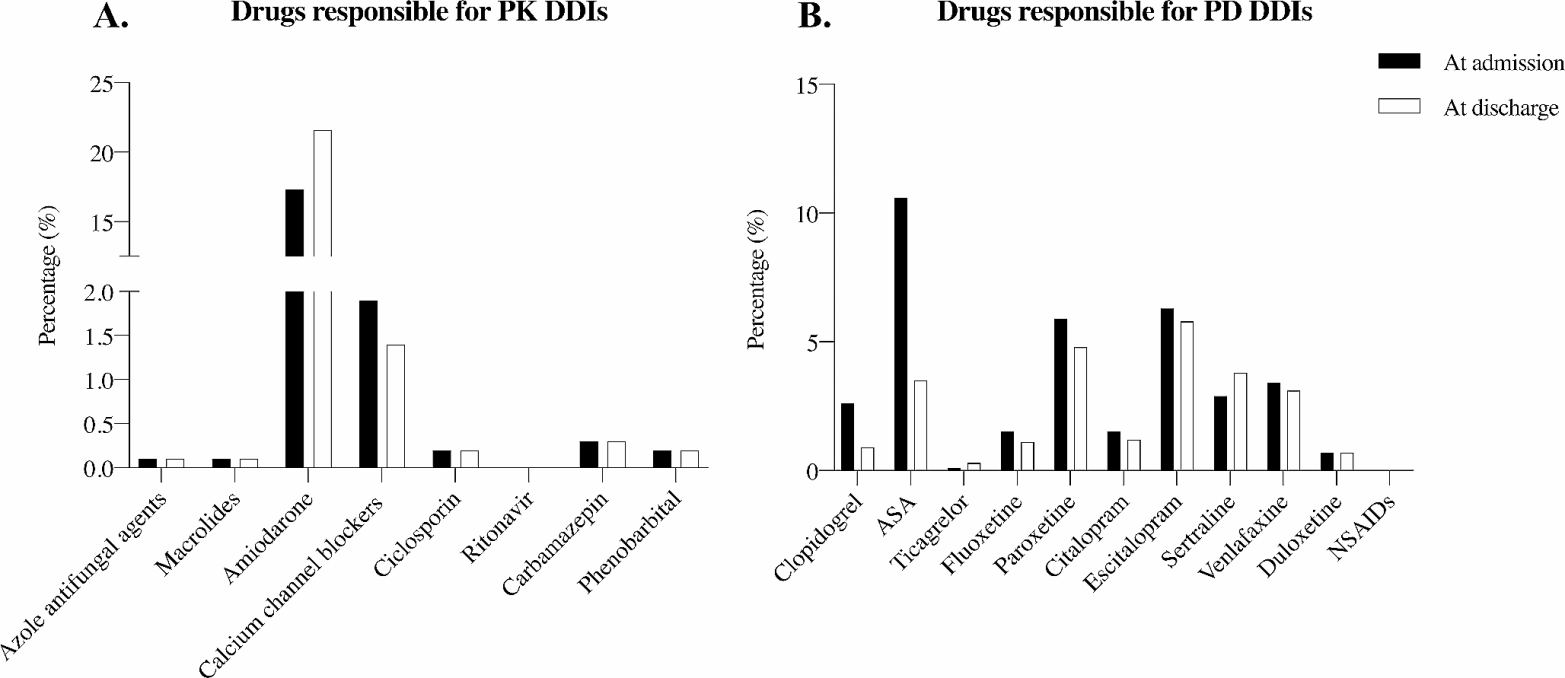
Distribution of drugs responsible for PK DDIs (**A**.) and for PD DDIs (**B**.). The data represent the percentage proportions of each drug prescribed in the study population at admission (black bar) and discharge (white bar) for each graph. Azole antifungals agents include ketoconazole, itraconazole, posaconazole and fluconazole. Calcium channel blockers include verapamil and diltiazem. Macrolides include erythromycin and clarithromycin. Abbreviations: ASA: Acetyl Salicylic Acid. CYP3A4: cytochrome P450 3A4. DDIs: drug-drug interactions. NSAIDS: Non-Steroidal Anti-Inflammatory Drugs. P-gp: P-glycoprotein. PD: pharmacodynamic

The drugs responsible for PK DDIs included 0.1% azole antifungals, 0.1% macrolides, 0.2% cyclosporine, 0.0% ritonavir, 0.3% carbamazepin, and 0.2% phenobarbital at admission and discharge (Fig. 2.**A**). Amiodarone was found in 17.3% of cases at admission and 21.6% of cases at discharge, whereas calcium channel blockers were present in 1.9% of cases at admission and 1.5% of cases at discharge (Fig. 2.**A**). PD DDIs were caused by 13.2% versus 4.4% of antiplatelet agents and 22.2% versus 20.5% of SRIs at admission and discharge (Fig. 2.**B**).

Of all the DDIs identified, the most common (> 5%) are listed in Table 3 at admission versus discharge. These included the association between apixaban and amiodarone (59.7% vs. 69.7%), rivaroxaban and amiodarone (26.0% vs. 21.8%), rivaroxaban and calcium channel blockers (5. 2% at admission), apixaban and SRIs (55.3% vs. 66.3%), rivaroxaban and SRIs (27.4% vs. 23.9%), apixaban and antiplatelet agents (12.6% vs. 10.3%), and rivaroxaban and antiplatelet agents (6.8% at admission).

**Table 3:** Most common (> 5%) PK DDIs (CYP3A4 and/or P-gp interactors) and PD DDIs

	At admission	At discharge
**PK DDIs**		
Apixaban - amiodarone	92 (59.7)	131 (69.7)
Rivaroxaban - amiodarone	40 (26.0)	41 (21.8)
Rivaroxaban – calcium channels blockers	8 (5.2)	-
**PD DDIs**		
Apixaban – SRI	105 (55.3)	122 (66.3)
Rivaroxaban – SRI	52 (27.4)	44 (23.9)
Apixaban - antiplatelet agents	24 (12.6)	19 (10.3)
Rivaroxaban - antiplatelets agents	13 (6.8)	-

The data presented as number (percentage)

Calcium channel blockers include verapamil and diltiazem. Serotonin uptake inhibitors include fluoxetine, paroxetine, citalopram, escitalopram, sertraline, venlafaxine, duloxetine. Antiplatelet agents include acetyl salicylic acid, clopidogrel and ticagrelor

Abbreviations: DDIs: drug-drug interactions. CYP3A4: cytochrome P450 3A4. PD: pharmacodynamic. P-gp: P-glycoprotein. PK: pharmacokinetic. SRI: serotonin re-uptake inhibitors

### Factors associated with PK and/or PD DDIs

In this section, only the adjusted odds ratios that are significant are detailed, but all the odds ratios obtained in univariate or multivariate analyses are presented in Table 4. Factors associated with the risk of PK and/or PD DDIs were bleeding history (ORa 1.74 95% CI 1.13–2.68), number of drugs > 6 (ORa 2.54, 95% CI 1.88–3.46) and reduced dose (ORa 0.39, 0.28–0.54) at admission (Table 4). There was a simple trend between age and the risk of PK and/or PD DDIs at admission (ORa 0.75, 95% CI 0.56–1.02, *p* = 0.06) (Table 4). At discharge, age > 87 years (ORa 0.74, 95% CI 0.55–0.99), creatinine > 88 µM (ORa 1.42, 95% CI 1.06–1.91), number of drugs > 6 (ORa 2.01, 95% CI 1.48–2.72) and reduced dose (ORa 0.41, 96% CI 0.30–0.57) were significantly associated with the risk of PK and/or PD DDIs (Table 4).

**Table 4:** Logistic regression analysis of factors associated with PK and/or PD DDIs

	At admission				At discharge			
	Univariate		Multivariate		Univariate		Multivariate	
	OR (95%CI)	*p*	ORa (95%CI)	*p*	OR (95%CI)	*p*	ORa (95%CI)	*p*
Sex (male)	1.15 (0.86–1.54)	ns	-	-	1.11 (0.83–1.48)	ns	-	-
Age > 87 years	0.60 (0.45–0.79)	***	0.75 (0.56–1.02)	ns	0.64 (0.48–0.83)	***	0.74 (0.55–0.99)	**
ADL > 5	1.02 (0.77–1.35)	ns	-	-	1.14 (0.87–1.50)	ns	-	-
CCI > 7	1.31 (0.98–1.73)	ns	-	-	0.97 (0.74–1.29)	ns	-	-
Length of stay > 15 days	-	-	-	-	0.94 (0.71–1.24)	ns	-	-
Cognitive impairment	1.13 (0.86–1.51)	ns	-	-	0.90 (0.69–1.19)	ns	-	-
Weight > 60 kg	0.96 (0.73–1.27)	ns	-	-	1.03 (0.78–1.35)	ns	-	-
Albumin > 34 g/L	1.03 (0.78–1.37)	ns	-	-	0.97 (0.74–1.28)	ns	-	-
Creatinine > 88 µM	1.46 (1.10–1.93)	**	1.25 (0.92–1.70)	ns	1.41 (1.07–1.85)	**	1.42 (1.06–1.91)	**
Bleeding history	1.84 (1.22–2.76)	**	1.74 (1.13–2.68)	**	0.83 (0.54–1.26)	ns	-	-
^#^N drugs > 6	2.81 (2.11–1.74)	****	2.54 (1.88–3.46)	****	2.61 (1.96–3.48)	****	2.01 (1.48–2.72)	****
Reduced dose	0.40 (0.29–0.54)	****	0.39 (0.28–0.54)	****	0.69 (0.52–0.91)	****	0.41 (0.30–0.57)	****

^#^The number of drugs was considered at admission for DDIs at admission and conversely

Continuous variables have been dichotomized below or above their mean/median value

*p* = *p*-value: ns > 0.05, * < 0.05, ** < 0.01, ***<0.001, ****<0.0001

Abbreviations: ADL: Activities of Daily Living. CCI: Charlson Comorbidity Index. CI: Confidence Interval. DDIs: Drug-Drug Interactions. N: number of. OR: Odds Ratio. ORa: Odds Ratio adjusted. PD: Pharmacodynamic. PK: Pharmacokinetic

## Discussion

The aim of this study was to assess the prevalence and risk factors associated with PK DDIs involving CYP3A4 and/or P-gp or PD DDIs in older adults treated with DOACs. This study included 909 hospitalizations between January 1, 2018, and December 31, 2022 of patients with an average age of 87 years. Amiodarone and calcium channel blockers for PK DDIs and SRIs and antiplatelet agents for PD DDIs were the most frequently used drugs. PK DDIs were 16.9% at admission versus 20.7% at discharge and PD DDIs were 20.9% at admission versus 20.2% at discharge. Bleeding history, number of drugs > 6, and reduced dose of DOACs were associated with the risk of PK and/or PD DDIs at admission. Age > 87 years, number of drugs > 6, and reduced dose of DOACs were associated with this risk at discharge.

Although from a single center, the population studied was representative of an acute geriatric unit in real life both in terms of age and geriatric parameters used in the usual way. Indeed, the decline in ADL indicating a loss of independence, CCI of 7 suggesting multiple comorbidities, cognitive impairment in over 50% of patients, and a mean GFR around 60 mL/min/1.73 m² are all characteristics found in older patients or associated with aging. There are very few missing data because it is routinely collected in this type of units. Approximately 70% of patients received a reduced dose of DOACs, which is expected for a geriatric population [22]. DOAC dosages are adjusted for advanced age, low body weight, and impaired renal function, which are particularly impacted in older adults because of renal aging and the risk of malnutrition [23, 24]. Moreover, the prevalence of AF in octogenarians is high [7]. This partly explains the prevalence of CYP3A4 and/or P-gp inhibitors, since amiodarone belongs to this category of prescription.

The prevalence of PK DDIs was approximately 17% at admission and increased by 3–4% at discharge, with amiodarone prescription slightly increasing. DDIs involving strong inhibitors of CYP3A4 and/or P-gp, such as azole antifungal agents, macrolides, or ciclosporins, are anecdotal because there are few indications justifying their prescription or because clinicians help to minimize the risk of DDIs by limiting their prescription. Drugs responsible for PD DDIs were approximately 21% at admission and 20% at discharge, which were overrepresented by SRI prescriptions. The high proportion of patients suffering from cognitive impairment in this study (approximately 58%) could explain the high prescription of antidepressants due to the close links between cognitive impairment and depressive syndrome, particularly in older patients [25].

In this study, the impact of hospitalization on the prevalence of DDIs was small; therefore, it would be interesting to measure the role of outpatient medical follow-up on this prevalence. The drugs with the greatest impact on the frequency of prescription during hospitalization were amiodarone for PK DDIs and clopidogrel and acetyl salicylic acid (ASA) for PD DDIs. The increased prevalence of amiodarone was probably driven by the goal of cardiac rhythm control in the context of AF decompensation; however, the reason for hospitalization was not reported. The decrease in antiplatelet agent prescriptions is probably due to a reassessment of treatment by the clinician during hospitalization. Indeed, some patients may have coronary artery disease associated with AF, and the duration of dual antithrombotic therapy had not been reviewed before hospitalization [26]. However, a combination of DOAC and antiplatelet agent is sometimes necessary in certain clinical situations, such as coronary pathology.

In the present study, age and reduced dose of DOACs were inversely associated with the risk of DDIs, probably due to the particular attention given to this type of patient. In fact, there are indications for reduction in the dose of DOACs according to age, weight, and renal function of patients, reflecting a certain underlying frailty [27]. It is therefore possible that clinicians censor prescribing for these patients, resulting in fewer DDIs. Bleeding history was associated with DDI risk at admission but not at discharge. Given the design of this study, it was not possible to determine whether the bleeding history was due to DDIs received at the time of hospital admission. However, the fact that it was not more associated with discharge from the hospital probably means that this parameter is not correctly or wrongly taken into account in the patient’s prescription involving DDIs. In the literature, the prevalence of PK DDIs is approximately 45%, with a linear increase in risk with age [28–30]. Gallo et al. [6] focused on PK DDIs involving CYP3A4 and P-gp in older adults because of the large number of drugs involved. Their aim was not to target a particular drug class, but to identify the most common DDIs in a sample of older adults and the parameters associated with these DDIs as exhaustively as possible [6]. Polypharmacy, defined as number of drugs greater than 6 (mean number of drugs in the study population), was found to be the factor with the strongest expected association [6], irrespective of the study or ours. Overall, PK DDIs do not always have a clinical impact, and statin prescriptions are particularly prevalent and represent a known risk of side effects among drugs particularly affected by DDIs in their study [6, 31]. According to some authors, statins are responsible for DDIs with DOACs; however, we have taken care not to include statins in our analysis because there are no studies on the PK impact of their co-administration with DOACs. DOACs also represent a risk of PK DDIs involving CYP3A4 and P-gp, which may be responsible for bleeding and are frequently used in older adults [7–11]. Their prevalence was probably underrepresented in the Gallo et al. study, prompting us to focus on this therapeutic class specifically [6].

The methodological choices regarding the statistical analysis were inspired by the study by Gallo et al. [6], which aimed to “reproduce” the aim but target PK DDIs involving DOACs. All variables were categorically analyzed based on the means or medians of the continuous variables. PD DDIs were also analyzed only in our study because of their clinical interest in the study of DOACs. In addition, Another notable difference in our study was the repeated inclusion of certain patients. This choice is justified by frequent changes in the treatment of older adults in terms of deprescription and therapeutic reinforcement associated with hospitalization [32]. AF is frequently associated with heart failure with a significant risk of cardiac decompensation at one year, which may lead to therapeutic modifications [33]. Moreover, with the exception of a slight increase in power, this methodological strategy probably had a minor influence because only 46 patients were included multiple times in this study.

In this study, the PK DDIs that could influence the DOACs’ PK parameters (Cmax, Tmax, Aera Under the Curve - AUC) were considered. However, not all PK DDIs have a well-defined clinical impact [14–17]. The European Medicines Agency does not recommend any dose adjustment except dabigatran when verapamil is co-administered [34]. On the other hand, the Food and Drugs Administration (FDA) recommends dose adjustment of apixaban, rivaroxaban, and dabigatran in case of co-administration of strong CYP3A4 and/or P-gp inhibitors [34]. In addition, the FDA mentions an “avoid use"of moderate CYP3A4 and/or P-gp inhibitor co-administration of these three DOACs [16]. In the present study, amiodarone, a CYP3A4 and/or P-gp inhibitor, was overrepresented in this category. The clinical impact of co-administration of amiodarone and DOACs, particularly on the risk of bleeding, is debated because clinical studies are contradictory [35–37]. SRIs were also well represented in this study. Although most SRIs are CYP3A4 inhibitors, they were counted in PD DDIs because of their interference with platelet function, which may be responsible for bleeding [16].

The main limitation of the present study is the lack of evaluation of the clinical or hemostatic consequences of DDIs. However, DDIs are not always responsible for clinical consequences, such as side effects or lack of therapeutic effect. In the present study, the exploratory method was limited to assessing the prevalence of DDIs and associated risk factors in a population of older patients to highlight this issue. The ADAGE study provides original data in patients over 80 years of age receiving DOACs, showing wide interindividual variability in plasma concentrations and thrombin generation parameters, considering an exhaustive survey of CYP3A4 and/or P-gp interactors [38]. However, there is still a need for large-scale studies focusing specifically on this patient population to measure the clinical impact. In the present study, history of bleeding was reported mainly to measure an association with the risk of DDI without being able to establish chronologically whether there was a link between the presence of DDI and the onset of bleeding. Therefore, the present study identified the most frequent PK or PD DDIs in a population of older subjects treated with DOACs. To date, there are no recommendations in Europe on dosage adjustments or specific monitoring when DOACs are prescribed. The majority of hospital prescription software contains prescription aids with DDI alerts. However, these alerts are not always relevant. Therefore, it is important for clinicians to be aware of the DDIs impacting the PK or PD of DOACs to react and establish diagnostics in case of adverse events such as bleeding or thrombosis.

## Conclusion

Polypharmacy is a particularly frequent situation in the older population and considerably increases the iatrogenic risk in the hospital. DOACs are frequently prescribed for older patients and are subject to several DDIs. These DDIs may lead to dramatic clinical situations, such as major bleeding. Our study highlighted the most frequent DDIs in a real-life population of elderly individuals. In addition, it identified certain risk factors, such as bleeding history and polypharmacy, which warrant closer monitoring of DOACs. On the other hand, age and reduced doses of DOACs were inversely associated with the risk of DDIs, probably due to prescription censorship. There is a need for more extensive studies that consider the impact of these DDIs specifically on older populations. Although there is no specific recommendation, prescribing DOACs should encourage clinicians to carefully assess DDIs because the risk of adverse effects is likely to be higher in older adults than in younger patients.

## Data Availability

The data presented in this study are available on request from the corresponding author.
